# Atypical Presentation of Atraumatic Spinal Subdural Hematoma Associated with Warfarin: A Case Report and Review of the Literature

**DOI:** 10.1155/2019/4037916

**Published:** 2019-05-20

**Authors:** Abdul Rehman Arain, Muhammad Moral, Saadia Shams, Khusboo Desai, Khunwar Kalsa

**Affiliations:** ^1^Department of Orthopedics, Albany Medical College, 1267 Washington Ave Suite 202, Albany, NY 12206, USA; ^2^Rawalpindi Medical College, Pakistan

## Abstract

Nontraumatic spinal subdural hematomas secondary to anticoagulants are remarkably rare. A case of spontaneous, atraumatic subdural hematoma involving the thoracic region in an 80-year-old woman on warfarin is reported. The patient presented with gross motor and sensory loss, delayed onset of incontinence, and no other symptoms. An MRI suggested an epidural hematoma concentrated around the T4-T9 levels. She was taken emergently to the OR approximately 30 hours after the initial onset of symptoms for a T3-T11 laminectomy. No epidural hematoma was noted. However, discoloration and bulging of the thecal sac were noted, and the dura was incised longitudinally from T2 to T10 revealing an expansive jelly-like blood clot which was evacuated. Postoperatively, the patient had regained 1/2 sensory function in the bilateral lower extremities. At the 2-week mark, the patient was still incontinent and showed 2/2 sensory and 2/5 motor functions in select muscle groups in her bilateral lower extremities. Completely nontraumatic, spontaneous subdural hematomas of the spine are very rare, and early surgical decompression within 24 hours from symptom onset may allow neurological recovery. Large extensive laminectomies up to 10 thoracic levels have been shown to be safe and effective in a few cases, including our case.

## 1. Introduction

Spontaneous epidural hematomas of the spine are a rare entity; they are reported in the literature with approximately 600 reported cases [1, 2]. Spontaneous spinal subdural hematomas (SSDH) are extremely rare with only a few cases reported in the literature. Patients usually present with acute low back pain or radicular pain, followed by rapid neurological deterioration.

Emergent surgical decompression is the standard of care for both epidural and subdural hematomas.

Most reported cases of SSDH involve patients on anticoagulants with precipitating events prior to presentation such as a sudden cough, recent lumbar punctures, and a recent increase in physical activities, AV malformation, and neoplasms [1, 3, 4]. To our knowledge, this is the 5^th^ reported case of SSDH involving warfarin [5, 6] and the only case completely absent of accompanying symptoms such as back pain, headache, nausea, and vomiting.

## 2. Case Presentation

**Figure 1:**
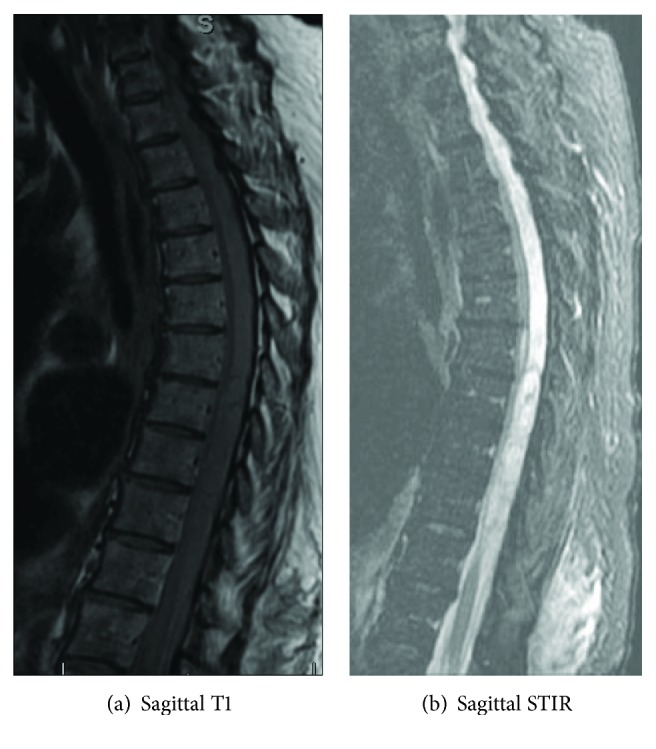
Sagittal T1 (a) and STIR (b) images of the thoracolumbar spine. A large hyperintense signal from the proximal thoracic to the lumbar spine can be seen on the STIR image.

We report on an 80-year-old Caucasian woman on warfarin for atrial fibrillation who sustained a spontaneous, atraumatic, spinal subdural hematoma in the thoracic region. The patient awoke in the morning to use the bathroom. Approximately 1 hr after returning to her bed, she was unable to move bilateral lower extremities and was incontinent. She reported no back pain, headache, nausea, vomiting, or any constitutional symptoms. She was transferred to an outside hospital and underwent an MRI, which as read by an attending senior radiologist who suggested an epidural hematoma concentrated around the T4-T9 levels (Figures 1(a) and 1(b)). She was reversed for an INR of 3.6 and then transferred to Albany Medical Center for further management. On examination, the patient was comfortable without any pain. She had a loss of bowel and bladder function and had no sensory or motor function below T5. The patient was seen by a fellowship-trained orthopaedic spine surgeon, and her spinal cord injury was classified as a T5 ASIA impairment scale A.

She was taken emergently to the OR approximately 30 hours after the initial onset of symptoms. After a T3-T11 laminectomy, the spinal cord was fully visible, but no epidural hematoma was noted. However, discoloration and bulging of the thecal sac were noted, and the dura was incised longitudinally from T2 to T10 revealing an expansive jelly-like blood clot. The hematoma was evacuated, and the dura closed with a 4′0-NUROLON.

**Figure 2:**
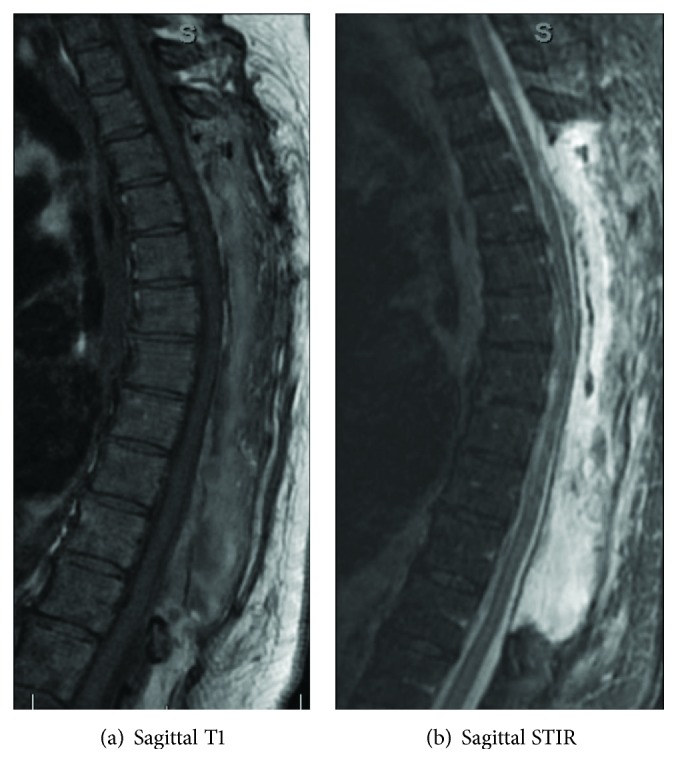
Sagittal T1 (a) and STIR (b) postoperative MRI images of the thoracolumbar spine showing a large multilevel thoracolumbar laminectomy. Postoperative fluid collections are demonstrated on the STIR image.

Postoperatively, the patient had regained 1/2 sensory function in the bilateral lower extremities. At the 2-week mark, the patient was still incontinent, showed 2/2 sensory and 2/5 motor functions in select muscle groups in her bilateral lower extremities. The patient's spinal cord injury was classified as an L2 ASIA impairment scale C. An MRI demonstrated a multilevel decompressive thoracolumbar laminectomy (Figures 2(a) and 2(b)), and the patient was discharged to a rehabilitation facility. At the two-month follow-up period, the patient had transitioned to a long-term nursing care facility and her neurological status remained unchanged.

## 3. Discussion

The true pathophysiology of spontaneous subdural hematomas is limited to two theories. Minor trauma, or increased intra-abdominal/thoracic pressure, may be associated with the rupture of valveless radiculomedullary veins in the subarachnoid space [7]. Alternatively, similar mechanisms may damage the few thin, delicate extra-arachnoidal vessels located on the inner dural surface, which then break through the arachnoid into the subarachnoid space leading to subdural hematomas [8, 9].

The classic clinical presentation of anticoagulant-associated spontaneous subdural hematoma is severe back pain, neck pain, or radicular pain, often preceding neurological demise [10]. Signs of increased intracranial pressure such as headache, nausea, and giddiness is also frequently reported during this prodromal phase [11]. In all reported cases, neurological symptoms of cord compression were rapidly progressive, and within a few hours of initial symptom onset, most patients had a complete loss of bowel and bladder function. In contrast to previously reported cases, our patient had no pain or prodromal symptoms. The patient's neurological symptoms included incontinence with complete loss of motor and sensory functions, which were all sudden and not progressive. Therefore, clinicians should be aware that even in the absence of classic prodromal symptoms, a spinal hemorrhage should be on the list of differentials for any patient on anticoagulants presenting with rapid neurological compromise.

Although MRI findings in spinal hematomas can vary based on the clot, age, and oxygenation, it remains the gold standard for diagnosis. Within the first 24 h after symptom onset, the hematoma shows isointensity on T1WI and hyperintensity on T2WI. After 24 h, it appears as a high signal on T1WI and as a low signal on T2WI. On sagittal images, subdural hematomas appear clumped and loculated with concave delineation. In contrast, epidural hematomas usually have a convex shape. However, the differentiation of epidural and subdural hematomas with an MRI is challenging. Similar to our case, Badge and Chan describe a case in which an initial MRI showing an epidural hematoma was realized to be a subdural hematoma intraoperatively [11]. In both cases, the decision to incise the dura, evacuate the hematoma, and repair the dura was made intraoperatively. It is difficult to assess if these unexpected intraoperative obstacles had any negative effects on the overall outcome for both patients. Due to the possible conflict between preoperative imaging and intraoperative findings, it is reasonable that any surgeon attempting to evacuate an epidural hematoma be prepared with appropriate tools and surgical backup, if needed, to perform intradural work in case a subdural hematoma is encountered.

Acute surgical decompression and discontinuation of anticoagulants is the standard of care in any patient on anticoagulants who is suspected of having a subdural hematoma [12–15]. Studies involving subdural and epidural hematomas of various etiologies suggest optimum results when surgical decompression occurs within 24 hours from the onset of symptoms [13, 16]. Large multilevel laminectomies for spinal hematoma management have shown to be safe and effective based on the limited literature available. Our case, a 9-level thoracic laminectomy is the second largest laminectomy reported for treating a spontaneous subdural hematoma. Visocchi et al. describe a 10-level thoracic laminectomy for a subdural hematoma occurring a few weeks after a lumbar puncture in a patient who improved from an ASIA impairment scale A to an ASIA impairment scale D at the 36 mo mark [16].

In our case, surgical management was delayed secondary to workup at an outside hospital. The patient's benign symptomatology, with a complete lack of pain and discomfort, may have thwarted an immediate transfer to a level 1 trauma center. The patient was transferred after an MRI showed a spinal hematoma. Upon arrival at our institution, the patient had to be triaged through the emergency department, with multiple teams evaluating the patient prior to the spine surgery team, which further delayed surgical management. We believe streamlined transfers from outside hospitals with adequate communication between the transferring team and surgical team assuming care of the patient would be invaluable in optimizing time-sensitive surgical outcomes, such as those involving spinal hemorrhage.

## 4. Conclusion

Atraumatic subdural hematoma is a rare but devastating entity associated with rapid neurological compromise. MRI is the imaging of choice, but differentiation between an epidural and a subdural hematoma remains a challenge. Although subdural hematomas are not frequently reported in the literature, the actual incidence may be higher as these can be mistaken for epidural hematomas, are often diagnosed intraoperatively, and may go unreported. Therefore, clinicians should be prepared to tackle either entity prior to surgical management of any spinal hemorrhage. Early surgical decompression within 24 hours from symptom onset is ideal for optimum neurological recovery. Large extensive laminectomies up to 10 thoracic levels are safe and effective.
